# Key predictors of 30-day mortality in non-cardiac surgery: development, key risk factors, and calibration of a risk model

**DOI:** 10.3389/fmed.2026.1749782

**Published:** 2026-01-30

**Authors:** Mantana Saetang, Thitikan Kunapaisal, Sirinporn Limvatanalert, Khwanrut Sukitpaneenit, Khantaros Saelim, Dararat Yongsata, Orawan Muangsong, Mananya Bunkerd, Sopida Kampeng

**Affiliations:** Department of Anesthesiology, Faculty of Medicine, Prince of Songkla University, Songkhla, Thailand

**Keywords:** 30-day mortality, nomogram, non-cardiac surgery, perioperative complications, risk predictors

## Abstract

**Introduction:**

Thirty-day mortality remains a critical indicator of perioperative safety and quality of care in patients undergoing non-cardiac surgery. Identifying predictive factors for 30-day mortality is critical for crisis mitigation and improving patient outcomes. This study aimed to identify key predictors of 30-day mortality in non-cardiac surgery patients and develop a predictive model to aid clinical decision-making.

**Methods:**

This retrospective observational study analyzed data from patients aged ≥18 years who underwent non-cardiac surgery at a tertiary care hospital between January and August 2022. Data on demographics, comorbidities, intraoperative variables, and postoperative complications were collected. Multivariate logistic regression identified independent predictors of 30-day mortality, and a nomogram was constructed to facilitate individualized risk assessment.

**Results:**

Among 7,528 patients, 76 (1.0%) died within 30 days. Independent predictors of mortality included preoperative ventilatory support (odds ratio [OR] 8.76; 95% confidence interval [CI]: 3.58–21.42; *p* < 0.001), postoperative sepsis (OR 5.86; 95% CI: 2.23–15.37; *p* < 0.001), coma (OR 18.56; 95% CI: 6.81–50.55; *p* < 0.001), myocardial infarction (OR 26.35; 95% CI: 4.46–155.63; *p* < 0.001), and cardiac arrest (OR 57.9; 95% CI: 18.22–184.05; *p* < 0.001). The nomogram demonstrated good calibration at lower risk levels, with slight overestimation at higher probabilities. The area under the receiver operating characteristic curve indicated excellent discrimination.

**Discussion:**

The developed nomogram serves as a useful tool for perioperative risk stratification in non-cardiac surgery. Incorporating both preoperative and postoperative factors, aids in early identification of high-risk patients and supports targeted clinical interventions. External validation and refinement of the model are recommended for broader applicability.

## 1 Introduction

Thirty-day mortality remains a key indicator of perioperative safety and quality of care in patients undergoing non-cardiac surgery. Despite advances in surgical techniques, anesthesia, and perioperative management, postoperative mortality continues to pose a significant clinical burden, particularly among older patients and those with multiple comorbidities. Understanding perioperative factors associated with 30-day mortality is therefore essential to improve risk stratification, guide clinical decision-making, and optimize perioperative management strategies.

Among the contributors to perioperative mortality, cardiac arrest represents one of the most catastrophic events. Although relatively uncommon occurring in approximately 7–30 per 10,000 non-cardiac surgical cases (1, 2). Intraoperative cardiac arrest is associated with extremely poor outcomes. Previous studies report 30-day mortality rates of 60–80% following perioperative cardiac arrest, particularly in elderly patients and those with significant comorbidities (1, 3). Complications following cardiac arrest, including septicemia, ventilator dependence, renal impairment, and major bleeding, further contribute to low survival rates (4, 5).

In addition to cardiac arrest, multiple perioperative factors have been shown to influence 30-day mortality. These include advanced age, higher American Society of Anesthesiologists (ASA) physical status classification, reduced functional capacity, clinical sepsis, emergency surgery, and end-stage renal disease (5–8). Many of these factors are also associated with an increased risk of intraoperative and postoperative cardiac arrest, highlighting the overlapping and cumulative nature of perioperative risk. Recognition of these predictors may allow anesthesiologists and surgeons to identify high-risk patients, implement targeted interventions, and potentially reduce postoperative mortality.

While prior studies have extensively examined risk factors for intraoperative cardiac arrest and its associated outcomes, there remains limited evidence on comprehensive predictive models specifically designed to estimate 30-day mortality in patients undergoing non-cardiac surgery. Accordingly, this study aimed to identify key perioperative predictors of 30-day mortality and to develop a predictive scoring system to support perioperative risk stratification in this patient population.

## 2 Materials and methods

### 2.1 Institutional review board approval

This study (REC.65-131-8-1) was approved by the Institutional Ethics Committee of the Faculty of Medicine, Prince of Songkla University, Songkhla, Thailand on April 22, 2022. The requirement for written informed consent was waived because this study utilized only retrospective clinical data collected during routine practice.

### 2.2 Study design and setting

**Figure 1:**
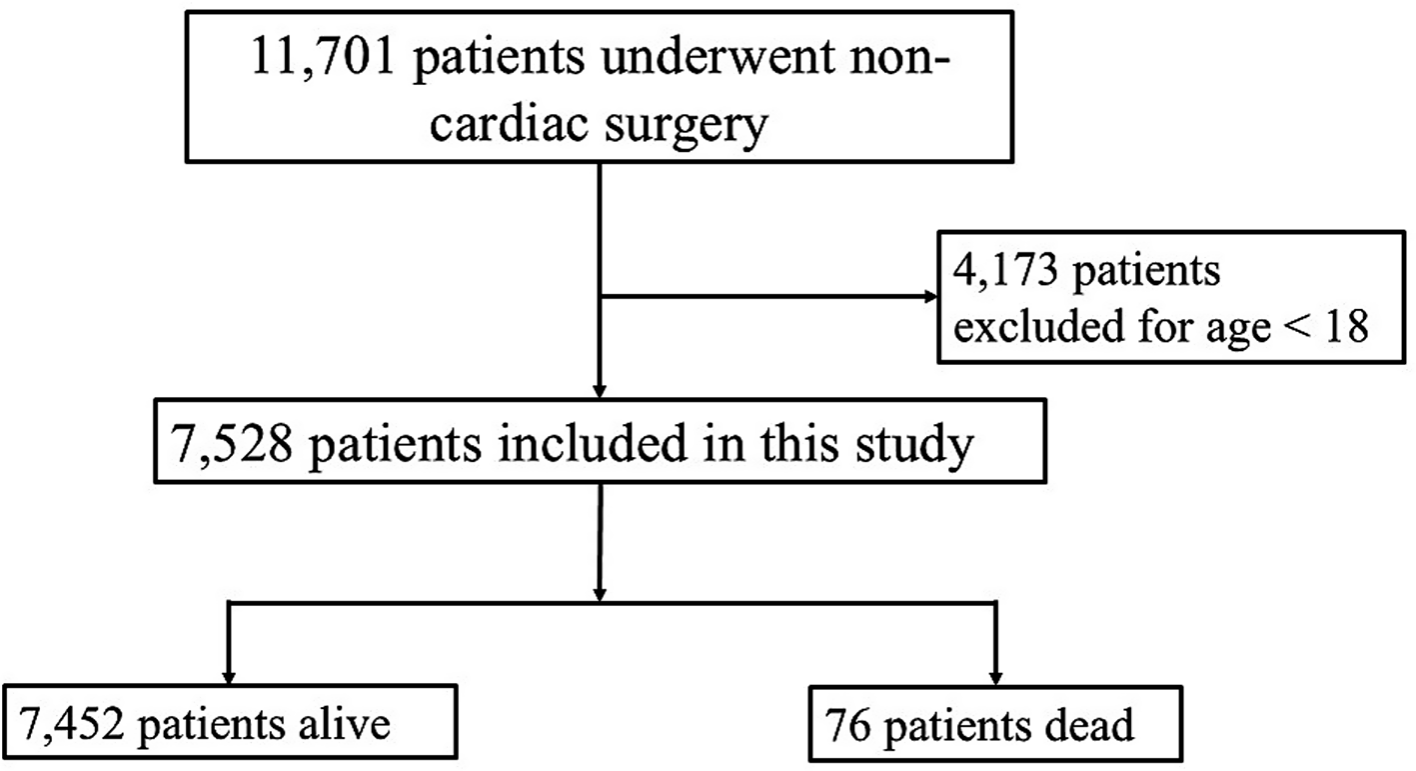
Flow chart for the study.

This retrospective observational study was conducted at Songklanagarind Hospital, a tertiary care facility in Southern Thailand. The study included all patients at the center from January 2022 to August 2022 who met the following criteria: aged ≥18 years and underwent non-cardiac surgery. Patients <18 years who underwent non-cardiac surgery were excluded to maintain a focus on identifying associations between predictive factors and 30-day mortality in the target population (Figure 1). The data for the study were collected between September 1, 2022, and September 30, 2023.

### 2.3 Data collection

Data on demographics (age, sex, body mass index), ASA physical status classification, type of surgery, patient comorbidities, and anesthesia techniques were collected from electronic medical and anesthesia records. The primary outcome was 30-day mortality, defined as all-cause death occurring at any time from the start of surgery up to 30 days postoperatively. Intraoperative mortality was not analyzed as a separate outcome but was included as a subset of the 30-day mortality endpoint, ensuring that all deaths occurring during surgery and the postoperative period were captured within a single, clinically meaningful outcome measure.

Sepsis was defined according to clinical documentation consistent with Sepsis-3 criteria, including suspected or confirmed infection with associated organ dysfunction occurring within the peri- or post-operative period. Myocardial infarction was defined as new ischemic symptoms, electrocardiographic changes, or elevated cardiac biomarkers documented by the treating physician within 30 days after surgery. Renal failure was defined as acute kidney injury requiring renal replacement therapy or a documented diagnosis of acute renal failure during hospitalization. Altered consciousness (coma) was defined as a documented Glasgow Coma Scale score ≤8.

### 2.4 Statistical analysis

The data were analyzed using R studio version 4.3.1 to evaluate the differences between survivors and non-survivors within 30 days of surgery. Continuous variables are summarized as means with standard deviations for normally distributed data or medians with interquartile ranges (IQRs) for non-normally distributed data. Student’s *t*-test was employed to compare normally distributed continuous variables between groups, whereas the Wilcoxon rank-sum test was applied for non-normal distributed variables.

Categorical variables are expressed as frequencies and percentages. The chi-squared test was used to compare proportions for variables with large sample sizes and sufficient expected frequencies. For categorical variables with small sample sizes or when expected cell frequencies were <5, Fisher’s exact test was applied for validity.

Univariate analyses were conducted to identify factors associated with 30-day mortality. Variables with a *p*-value <0.20 in univariate analysis were included in a multivariate logistic regression model to identify independent predictors of mortality. Given that there were 76 deaths, we ensured that only 7–8 variables were included in the final model to maintain statistical validity and avoid overfitting. The selected variables were determined based on clinical relevance and statistical significance. Logistic regression analysis results were reported as odds ratios (ORs) with 95% confidence intervals (CIs). Statistical significance was defined as a *p*-value < 0.05.

The predictive performance of the model was evaluated using a calibration plot to compare the predicted probabilities of mortality with observed outcomes. Bootstrapping with 1,000 resamples was conducted to correct for optimism and overfitting in the model. The model’s discrimination ability was evaluated using receiver operating characteristic (ROC), which quantified its capacity to distinguish between survivors and non-survivors.

This comprehensive statistical approach ensured robust analysis, accounting for data characteristics and providing reliable insights into the factors influencing 30-day mortality in patients undergoing non-cardiac surgery.

## 3 Results

### 3.1 Baseline patient characteristics and mortality rates

A total of 7,528 patients were included in the study, of whom 76 (1.0%) experienced 30-day mortality. Patients who died were significantly older, with a median age of 66 years (IQR 49–73.2), compared to 53 years (IQR 36–66) for survivors (*p* < 0.001). A higher proportion of patients in the mortality group had an ASA classification of 3, 4, 5 accounted for 57.9, 23.7, and 10.5% of deaths, respectively, (*p* < 0.001). Emergency surgeries were more common in the mortality group (59.2%) than in survivors (23.0%, *p* < 0.001). Key preoperative factors significantly associated with higher mortality are summarized in Table 1.

**Table 1:** Baseline characteristics of patients undergoing non-cardiac surgery categorized by 30-day mortality.

Characteristics	Alive (*N* = 7,452)	Death (*N* = 76)	*p*-value
Male, *n* (%)	2,933 (39.4)	43 (56.6)	0.003
Age, median (IQR)	53 (36, 66)	66 (49, 73.2)	<0.001
Weight, median (IQR)	62.3 (54.2, 72)	59.5 (50, 70)	0.005
Height, median (IQR)	160 (155, 165)	160.5 (155, 170)	0.198
ASA classification, *n* (%)			<0.001
1	254 (3.4)	0 (0)	
2	4,727 (63.5)	6 (7.9)	
3	2,369 (31.8)	44 (57.9)	
4	90 (1.2)	18 (23.7)	
5	9 (0.1)	8 (10.5)	
Case type, *n* (%)			<0.001
Elective	5,605 (75.3)	31 (40.8)	
Emergency	1,715 (23)	45 (59.2)	
OPD	124 (1.7)	0 (0)	
History of ischemic heart disease, *n* (%)	305 (4.1)	8 (10.5)	0.012
History of congestive heart failure, *n* (%)	32 (0.4)	1 (1.3)	0.771
History of peripheral arterial disease, *n* (%)	113 (1.5)	3 (3.9)	0.214
History of arrhythmia, *n* (%)	275 (3.7)	16 (21.1)	<0.001
Smoking, *n* (%)	1,126 (15.1)	24 (32)	<0.001
Diabetic mellitus, *n* (%)	1,265 (17)	20 (26.7)	0.039
History of cerebrovascular disease, *n* (%)			<0.001
Fully recovered	235 (3.2)	3 (4)	
Partially recovered	196 (2.6)	10 (13.3)	
History of cancer, *n* (%)	1,662 (22.3)	27 (36)	0.007
On ventilator, *n* (%)	165 (2.2)	43 (56.6)	<0.001
Preoperative sepsis, *n* (%)	111 (1.5)	23 (30.3)	<0.001
Altered consciousness, *n* (%)	113 (1.5)	26 (34.2)	<0.001
Preoperative hyperthermia, *n* (%)	470 (6.4)	37 (51.4)	<0.001
Preoperative hypertensive emergency, *n* (%)	81 (1.1)	5 (6.7)	<0.001
Preoperative use of inotropes, *n* (%)	49 (0.7)	20 (26.7)	<0.001
Preoperative red blood cell transfusion, *n* (%)	157 (2.1)	26 (34.7)	<0.001
Preoperative hyperglycemia, *n* (%)	227 (8.1)	15 (28.8)	<0.001
Preoperative use of acetaminophen, *n* (%)	2,370 (31.8)	14 (18.4)	0.018
Preoperative hematocrit, median (IQR)	37.5 (33.8, 40.8)	30.4 (26.2, 37.3)	<0.001
Preoperative platelet count, median (IQR)	254,000 (201,000, 311,000)	233,000 (148,000, 305,250)	0.148
Creatinine, median (IQR)	0.7 (0.5, 0.9)	0.8 (0.6, 1.4)	<0.001
Albumin, median (IQR)	9 (4, 9)	3.3 (2.6, 5.6)	<0.001

IQR, interquartile range; ASA, American Association of Anesthesiologists; FiO_2_, fraction of inspired oxygen; INR, international normalized ratio; PTT, partial thromboplastin time; OPD, outpatient department.

### 3.2 Intraoperative factors

Mortality was associated with cases performed outside the OR (17.1% vs. 5.7%, *p* < 0.001), intraoperative tachycardia (34.2% vs. 16.5%, *p* < 0.001), and intraoperative cardiac arrest (5.3% vs. 0.1%, *p* < 0.001). Additionally, intraoperative hypotension (69.7% vs. 48.5%, *p* < 0.001) and the use of inotropic agents (60.5% vs. 16.3%, *p* < 0.001) were significantly more prevalent in the mortality group (Table 2).

**Table 2:** Intraoperative techniques and complications stratified by 30-day mortality.

Intraoperative techniques and complications	Alive (*N* = 7,452)	Death (*N* = 76)	*p* value
Intraoperative period			
Area, *n* (%)			<0.001
Operating room	7,029 (94.3)	63 (82.9)	
Remote area	423 (5.7)	13 (17.1)	
Duration of anesthetic time, median (IQR)	130 (75, 210)	115 (78.8, 205)	0.514
Invasive monitor, *n* (%)	1,284 (17.2)	43 (56.6)	<0.001
Arterial line, *n* (%)	1,275 (17.1)	43 (56.6)	<0.001
Central line, *n* (%)	213 (2.9)	20 (26.3)	<0.001
Endotracheal tube, *n* (%)	4,493 (60.3)	70 (92.1)	<0.001
Laryngeal mask airway, *n* (%)	74 (1)	0 (0)	0.773
Tracheostomy tube, *n* (%)	137 (1.8)	4 (5.3)	0.077
Intraoperative inotrope, *n* (%)	1,214 (16.3)	46 (60.5)	<0.001
Adrenaline, *n* (%)	26 (0.3)	12 (15.8)	<0.001
Dopamine, *n* (%)	15 (0.2)	7 (9.2)	<0.001
Levophed, *n* (%)	1,203 (16.1)	45 (59.2)	<0.001
Intraoperative hypotension, *n* (%)	3,613 (48.5)	53 (69.7)	<0.001
Intraoperative tachycardia, *n* (%)	1,227 (16.5)	26 (34.2)	<0.001
Intraoperative hyperglycemia, DTX > 180 mg/dL	169 (2.3)	9 (11.8)	<0.001
Duration of surgery, median (IQR)	90 (50, 165)	82.5 (50, 180)	0.883
Estimated blood loss, median (IQR)	50 (5, 300)	50 (10, 300)	0.404
Intraoperative cardiac arrest, *n* (%)	7 (0.1)	4 (5.3)	<0.001
Phase of arrest, *n* (%)			<0.001
Induction	3 (0)	2 (2.6)	
Anesthetic course	4 (0.1)	0 (0)	
Transport to recovery room	0 (0)	1 (1.3)	
Postoperative period			
Postoperative 24 h	0 (0)	1 (1.3)	
EKG in arrest, *n* (%)			<0.001
Asystole	2 (0)	2 (2.6)	
PEA	3 (0)	1 (1.3)	
VF/VT	2 (0)	1 (1.3)	
Cause of arrest, *n* (%)			<0.001
Bleeding	4 (0.1)	2 (2.6)	
Cardiac	2 (0)	1 (1.3)	
Hypoxia	0 (0)	1 (1.3)	
Other	1 (0)	0 (0)	
Intraoperative death, *n* (%)	4 (0.1)	1 (1.3)	0.044

IQR, interquartile range; N_2_O, nitrous oxide; DTX, dextrostrix; EKG, electrocardiogram; PEA, pulseless electrical activity; VF, ventricular fibrillation; VT, ventricular tachycardia.

### 3.3 Postoperative complications

Patients in the mortality group exhibited significantly higher rates of complications within 30 days. Sepsis (68.4%), pneumonia (55.3%), and renal failure (43.4%) were the most common complications among patients who died, all markedly elevated compared to the occurrence in survivors (*p* < 0.001; Table 3).

**Table 3:** Postoperative complications within 30 days stratified by mortality status.

Postoperative complications	Alive (*N* = 7,452)	Dead (*N* = 76)	*p*-value
Pneumonia	153 (2.1)	42 (55.3)	<0.001
Prolonged intubation	70 (0.9)	26 (34.2)	<0.001
Reintubation	38 (0.5)	6 (7.9)	<0.001
Coma	30 (0.4)	56 (73.7)	<0.001
Sepsis	141 (1.9)	52 (68.4)	<0.001
DVT/PE	32 (0.4)	10 (13.2)	<0.001
Renal failure	75 (1)	33 (43.4)	<0.001
Stroke	14 (0.2)	6 (7.9)	<0.001
Myocardial infarction	10 (0.1)	7 (9.2)	<0.001
Cardiac arrhythmia	43 (0.6)	21 (27.6)	<0.001
Cardiac arrest	8 (0.1)	50 (65.8)	<0.001
Reoperation	80 (1.1)	6 (7.9)	<0.001
Multiple organ failure	6 (0.1)	16 (21.1)	<0.001

Data are presented as numbers (%) unless otherwise indicated. DVT, deep vein thrombosis; PE, pulmonary embolism.

### 3.4 Multivariate analysis

Key predictors of 30-day mortality were identified through multivariate analysis, with the following variables showing strong associations: preoperative ventilatory support (OR 8.76; 95% CI: 3.58–21.42; *p* < 0.001), postoperative sepsis (OR 5.86; 95% CI: 2.23–15.37; *p* < 0.001), coma (OR 18.56; 95% CI: 6.81–50.55, *p* < 0.001), deep vein thrombosis or pulmonary embolism (OR 7.91; 95% CI: 1.8–34.7; *p* < 0.001), and myocardial infarction (OR 26.35; 95% CI: 4.46–155.63; *p* < 0.001) (Table 4).

**Table 4:** Univariate and multivariate analyses of factors associated with 30-day mortality.

Factors	Univariate		Multivariate	
	Odds ratio	*p*-value	Odds ratio	*p*-value
On ventilator	8.1 (2.86,22.95)	<0.001	8.76 (3.58, 21.42)	<0.001*
Preoperative sepsis	2.67 (0.73,9.77)	0.147		
Preoperative RBC transfusion	1.37 (0.74,2.55)	0.427		
Adrenaline	37.86 (7.32,195.92)	<0.001	27.32 (6.74, 110.67)	<0.001*
Intraoperative tachycardia	2.58 (1.05,6.35)	0.044		
Coma	18.52 (6.27,54.75)	<0.001	18.56 (6.81, 50.55)	<0.001*
Postoperative sepsis	6 (2.19, 16.39)	0.001	5.86 (2.23, 15.37)	<0.001*
DVT/PE	8.08 (1.89, 34.44)	0.008	7.91 (1.8, 34.7)	<0.001*
Myocardial infarction	38.22 (7.06, 207.04)	<0.001	26.35 (4.46, 155.63)	<0.001*
Cardiac arrest	64.47 (18.06, 230.12)	<0.001	57.9 (18.22, 184.05)	<0.001*

*Value is statistically significant. RBC, red blood cells; DVT, deep vein thrombosis; PE, pulmonary embolism.

Cardiac arrest emerged as the strongest independent predictor of 30-day mortality (OR 57.9; 95% CI: 18.22–184.05; *p* < 0.001). Additionally, the use of adrenaline during surgery markedly increased the risk (OR 27.32; 95% CI: 6.74–110.67; *p* < 0.001) (Table 4).

### 3.5 Nomogram and predictive model

**Figure 2:**
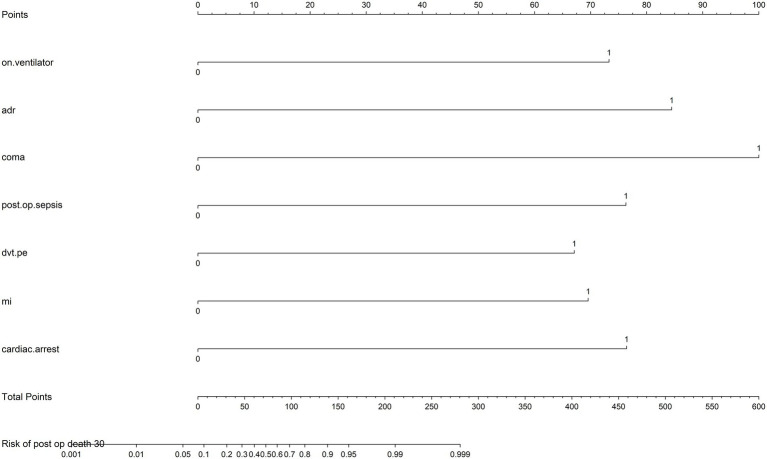
Nomogram for individualized risk assessment of 30-day mortality rates. The nomogram incorporates key predictors, including preoperative on ventilator support (on.ventilator), intraoperative use adrenaline (ADR), postoperative coma, postoperative sepsis (post.op.sepsis), deep vein thrombosis or pulmonary embolism (dvt.pe), myocardial infarction (MI), and cardiac arrest (cardiac.arrest). Each predictor is assigned a score based on its impact on risk, which can be summed to calculate the total points. The total points correspond to the predicted risk of 30-day mortality rates, displayed on the bottom scale.

A predictive nomogram was developed to incorporate key clinical variables, including preoperative ventilatory support, postoperative sepsis, myocardial infarction, and cardiac arrest. The tool allowed individualized risk assessment, with higher total scores corresponding to an increased probability of 30-day mortality (Figure 2).

**Figure 3:**
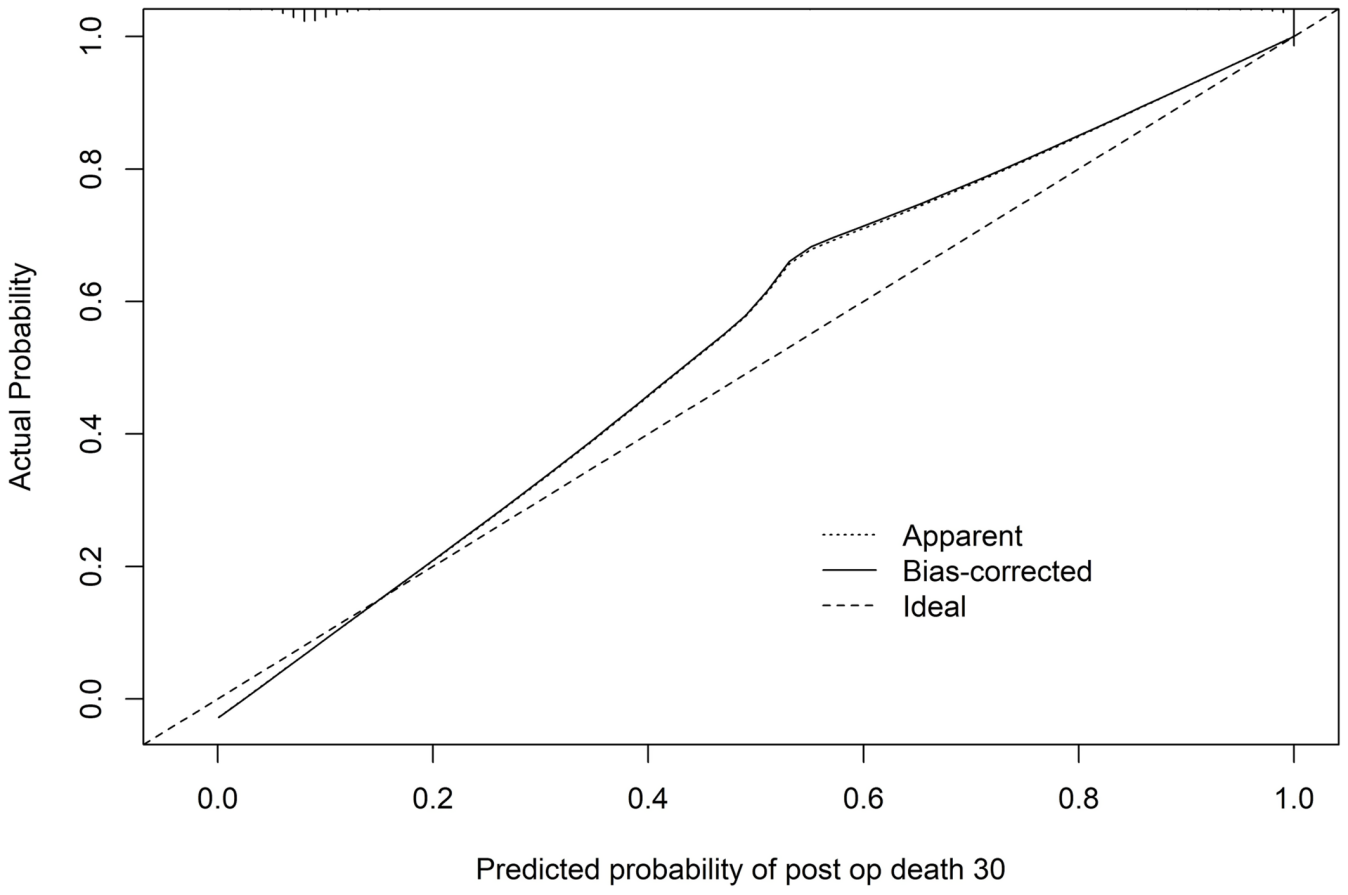
Calibration plot of the predictive nomogram for 30-day mortality risk. The calibration plot compares predicted probabilities from the nomogram with observed 30-day mortality rates. The dashed diagonal line represents the ideal calibration, while the solid line indicates the model’s performance. Overestimation is noted at higher probabilities. Mean absolute error = 0.013; mean squared error = 0.00061; quantile of absolute error = 0.021.

The nomogram’s performance was evaluated using calibration analysis, which demonstrated good accuracy at lower risk levels. However, a slight overestimation of mortality risk was observed for higher-risk probabilities (Figure 3). The model’s discriminatory ability was assessed using the area under the receiver operating characteristic (AUROC) curve as 0.96.

## 4 Discussion

This study evaluated factors associated with 30-day mortality in patients undergoing non-cardiac surgery and developed a predictive nomogram to support risk stratification. The findings highlight the importance of patient characteristics, intraoperative events, and postoperative complications contributing to mortality. These insights emphasize the critical impact of comprehensive perioperative management in improving surgical outcomes and optimizing risk stratification.

### 4.1 Key predictors of 30-day mortality

The multivariate analysis identified several key predictors of 30-day mortality, including preoperative ventilatory support, postoperative sepsis, coma, deep vein thrombosis, myocardial infarction, and cardiac arrest. Cardiac arrest demonstrated the highest OR, underscoring its critical impact on patient outcomes.

### 4.2 Preoperative factors

The association between preoperative factors, such as ventilatory support and preoperative sepsis, and increased mortality highlights the vulnerability of patients with compromised physiological reserves. Of these factors, preoperative ventilatory support emerged as the strongest independent predictor of mortality.

These findings are consistent with those of previous studies that identified preoperative physiological derangements as critical determinants of adverse outcomes. For instance, Kazaure et al. (4) reported that renal impairment, disseminated cancer, and preoperative sepsis are independent predictors of poor survival. Similarly, Kaiser et al. (5) reported that ASA classification, advanced age, functional status, SIRS/sepsis, and disseminated cancer were the most substantial determinants of 30-day mortality. In contrast, our study found that preoperative sepsis, while significant in univariate analysis, did not remain significant in multivariate models. This distinction indicates the nuanced interplay between preoperative factors and their relative contributions to mortality risk. Additionally, Nuttall et al. (9) identified sepsis as the leading cause of death within 30 days postoperatively, followed by malignancy, cardiovascular disease, neurological disease, and hemorrhage. Magor et al. (10) also observed that patients requiring unplanned postoperative ventilatory support had a significantly higher mortality rate, with an adjusted OR of 5.8 (95% CI: 3.8–8.8, *p* < 0.001), a finding consistent with our result. Collectively, these findings emphasize the relevance of early identification and targeted optimization of modifiable factors. For example, the prompt treatment of infections and the management of physiological derangements can significantly mitigate risks and improve patient survival.

### 4.3 Intraoperative factors

The intraoperative period presents several critical challenges, particularly factors like tachycardia and the use of inotropes, such as adrenaline, which are strongly associated with increased mortality. However, the role of tachycardia in perioperative outcomes remains complex and incompletely understood. Ruetzler et al. (11) found no significant relationship between various measures of tachycardia and composite outcomes of myocardial injury and death, a finding that aligns with our results. In contrast, an earlier retrospective analysis of 41,140 patients found that a heart rate of at least 90 beats per minute was independently associated with an increased risk of myocardial injury (adjusted OR 1.22; 95% CI: 1.06–1.39; *p* = 0.005) (12). Similarly, Jindawatthana et al. (13) demonstrated that preoperative vasopressor use was an independent risk factor significantly associated with 30-day mortality (adjusted relative risk 1.90; 95% CI: 1.08–3.32; *p* = 0.025), consistent with our findings.

Maintaining hemodynamic stability is paramount, as even transient disturbances can trigger adverse outcomes in high-risk patients. The strong association between cardiac arrest and mortality underscores the importance of the immediate recognition and management of perioperative crises, using standardized protocols and advanced monitoring technologies. These measures are essential to minimize intraoperative risks and improve surgical outcomes, particularly in vulnerable populations.

### 4.4 Postoperative complications

This study confirmed that complications such as sepsis, myocardial infarction, deep vein thrombosis, and altered consciousness are significant predictors of mortality. These findings align with those from previous studies demonstrate the critical role of postoperative complications in surgical mortality. For instance, Park et al. (14) reported five post-operative complications, myocardial injury after non-cardiac surgery [MINS], major bleeding, sepsis, stroke, and acute kidney injury requiring dialysis, as independent predictors of death. Similarly, Vascular Events in Noncardiac Surgery Patients Cohort Evaluation (VISION) Study Investigators et al. (15) identified three complications with the largest attributable fractions (AF) for mortality: major bleeding (15.6%; adjusted hazard ratio [HR] 2.6; 95% CI 2.2–3.1; AF 17.0%); MINS (13.0%; adjusted HR 2.2; 95% CI 1.9–2.6; AF 15.9%); and sepsis (4.5%; adjusted HR 5.6; 95% CI 4.6–6.8; AF 12.0%). Additionally, postoperative cardiac arrest is independently associated with worse survival, further emphasizing the significant impact of severe postoperative events on mortality outcomes (4).

Early recognition and aggressive management of postoperative complications are critical for improving patient outcomes. Timely interventions, such as the administration of antibiotics for sepsis, early mobilization to reduce the risk of thromboembolic events, and vigilant cardiac monitoring, play a pivotal role in mitigating these risks. The strong association between these complications and mortality underscores the importance of structured postoperative care protocols.

Overall, the identification of high-risk patients based on these predictors allows clinicians to implement targeted and timely interventions, ultimately improving survival outcomes and reducing the burden of postoperative complications.

### 4.5 Nomogram as a predictive tool

The nomogram is, therefore, best interpreted as a perioperative risk aggregation tool, reflecting the cumulative impact of preoperative vulnerability, intraoperative instability, and early postoperative complications, rather than a purely causal or preoperative prediction model. We acknowledge that several variables included in the model, such as intraoperative cardiac arrest, use of inotropic agents, and severe hypotension, may lie directly on the causal pathway to death. Their inclusion may enhance statistical discrimination while limiting the usefulness of the model for early risk prediction.

The nomogram developed in this study provides a practical framework for estimating individual 30-day mortality risk (Figure 2) once major perioperative events have occurred. By integrating key predictors, including ventilatory support, sepsis, myocardial infarction, and cardiac arrest, the model facilitates stratification of patients according to evolving perioperative risk. Although calibration was good at lower predicted risk levels, the model slightly overestimated mortality at higher probabilities (Figure 3), consistent with the inclusion of catastrophic perioperative events. Consequently, the nomogram should be applied cautiously and be primarily used to support perioperative clinical decision-making and resource allocation, rather than as a tool for early or preoperative prognostication.

### 4.6 Clinical implications

These findings highlight the need for a multidisciplinary approach to perioperative care. Addressing modifiable preoperative factors, maintaining intraoperative hemodynamic stability, and implementing rigorous postoperative monitoring are crucial strategies to reduce surgical mortality. Incorporating the nomogram into clinical workflows could further enhance the clinician’s ability to identify high-risk patients and effectively prioritize interventions.

### 4.7 Future directions

Future studies should focus on validating the nomogram in external cohorts to assess its generalizability. Additionally, advanced predictive modeling techniques such as machine learning could improve the model’s accuracy and applicability. Evaluating the impact of targeted interventions based on risk stratification would provide critical insights into improving perioperative care for high-risk patients.

### 4.8 Limitations

This study has certain limitations that should be considered when interpreting the results. First, the retrospective design may introduce selection and information biases, as the data were collected from past records without real-time verification. Second, the small number of intraoperative cardiac arrest cases limit the ability to construct a reliable predictive model for this specific outcome. As a result, the primary focus shifted to identifying predictors of 30-day mortality. Third, the findings are based on data from a single tertiary care hospital, which may affect the generalizability of the results to other settings. Lastly, although the model demonstrated strong discrimination, several of the included variables, such as intraoperative cardiac arrest, use of inotropic agents, and severe hypotension, lie on the causal pathway to death. Their inclusion likely inflated model performance and limits the usefulness of the nomogram as an early or purely preoperative prediction tool. As a result, the nomogram should be interpreted as a perioperative risk aggregation model rather than a causal predictor of mortality. Future research should aim for external validation of the nomogram in diverse populations and consider using advanced machine-learning techniques to improve the model’s predictive accuracy.

## 5 Conclusion

This study identifies critical predictors of 30-day mortality in non-cardiac surgery and provides a nomogram for risk stratification. While postoperative factors play an important role, the inclusion of preoperative predictors offers some early prognostic value. Further refinement and validation of the model will enhance its clinical applicability. Therefore, the nomogram is best interpreted as a dynamic perioperative risk aggregation tool rather than a purely preoperative prediction model.

## Data Availability

The original contributions presented in the study are included in the article/supplementary material, further inquiries can be directed to the corresponding author.
